# Evaluation neointimal coverage in patients with coronary artery aneurysm formation after drug-eluting stent implantation by optical coherence tomography

**DOI:** 10.1007/s10554-013-0282-y

**Published:** 2013-09-13

**Authors:** Tian Feng, Chen Yundai, Liu Hongbin, Chen Lian, Sun Zhijun, Guo Jun, Jin Qinhua, Zhang Tao

**Affiliations:** Department of Cardiology, Chinese PLA General Hospital, Beijing, 100853 China

**Keywords:** Drug-eluting stent, Coronary artery aneurysms, Optical coherence tomography

## Abstract

The neointimal coverage in patients with coronary artery aneurysms (CAA) formation after drug eluting stent (DES) implantation is not clear. Total of 175 patients who had been implanted DES were identified. Patients were divided into the CAA group (n = 31) and non-CAA group (n = 144) based on the results of the coronary angiography. The cardiac events including angina and acute myocardial infarction were noted, in addition, the neointimal thickness and the frequence of strut malapposition and strut uncoverage were noted. A greater proportion of incomplete neointimal coverage (17.17 vs. 1.9 %, *P* < 0.001) and malapposition struts (18.2 vs. 1.38%, *P* < 0.001) were observed in the CAA group. 8 patients in CAA group underwent OCT examination twice in the period of follow-up. The proportion of incomplete neointimal coverage increased significantly as compared the second OCT results with the first examination (18.45 vs. 2.66 %, *P* < 0.001). Hyperplasia neointimal desquamated from struts and acquired struts incomplete neointimal coverage were detected. Patients with CAA had a higher frequency of cardiac events including angina pectoris (25.81 vs. 6.25 %, *P* = 0.001) and acute myocardial infarction (9.68 vs. 0.13 %, *P* = 0.002) and thrombosis (16.13 vs. 0.69 %, *P* < 0.001). The longitudinal length of CAA in cardiac event group was significantly longer than no cardiac event group (20.0 ± 9.07 vs. 12.05 ± 5.38 mm, *P* = 0.005). CAA formation after DES implantation frequently associated with cardiac events as a result of stent malapposition and incomplete neointimal coverage. Acquired incomplete neointimal coverage associated with CAA formation.

## Introduction

Some studies have reported that coronary artery aneurysm (CAA) occurred in 0.3–6.0 % of patients after stent implantation [1, 2]. The potential causes were the delayed endothelialization, inflammatory, antimetabolite effect of the drug, hypersensitivity reactions to the drug and polymer [3–5]. Based on the follow-up intravascular ultrasound (IVUS) examination, CAA formation after DES implantation could enlarge the external elastic membrane and increase late stent malapposition [6, 7]. However, IVUS cannot assess the neointimal coverage after stent implantation because of its limited resolution. Therefore, the status of neointimal coverage in patients with CAA formation after DES implantation remains to be determined. Optical coherence tomography (OCT) is a new imaging modality with a higher resolution, approximately tenfold better than that of the IVUS image. OCT can assess vessel healing more clearly and accurately during the follow-up of DES compared with IVUS [8, 9]. The current study aims to assess the vessel healing in patients with CAA formation after DES implantation.

## Methods

### Study population

From June 2008 to August 2011, follow-up examinations were conducted on 1,160 patients who underwent percutaneous coronary intervention (PCI). The average period of follow-up was approximately (18.9 ± 13.1) months after PCI. CAA at the segment of stent implantation occurred in 56 patients. Total of 31 patients with CAA formation underwent OCT examination during follow-up. We labeled those 31 patients as CAA group (n = 31), and randomly chose 144 patients who underwent DES implantation into de novo lesions and underwent coronary angiography and OCT examination during follow-up without CAA formation . Those 144 patients were labled as non-CAA group (n = 144). The exclusion criteria included congestive heart failure, renal insufficiency (serum creatinine > 1.8 mg/dL), left main disease, target vessel diameter ≥4.0 mm, and history of revascularization. All patients provided written informed consent prior to coronary angiography and OCT examination.

### Coronary angiography and PCI

Coronary angiography was performed using a 6 French catheter through the femoral or radial artery. Heparin (100 U/Kg) was administered conventionally, and imaging was performed after administering 200 μg of intracoronary nitroglycerin. PCI was performed in a standard manner, and the lesion was completely covered by stents. The types of DES implantation included the Cypher stent (sirolimus-eluting stent, Cordis, USA), Endeavor stent (Zotarolimus-eluting stent, Medtronic, USA), Firebird stent (sirolimus-eluting stent, Microport, China), Partner stent (sirolimus-eluting stent, Lepu, China), and Excel stent (sirolimus-eluting stent, JW, China). The stents were successfully implanted without complication, and all patients were prescribed with oral aspirin (100 mg/d) in life and clopidogrel (75 mg/d) for at least 1 year. The patients underwent coronary angiography examination in the period of follow**-**up. Coronary angiograms (including baseline, intervention and follow-up) were analyzed by two doctors in the angiographic core laboratory who were blind to the research. QCA was performed with an automatic edge-detection system (MEDIS, CMS 4.0, Leiden, the Netherlands). CAA was defined as a localized angiographic dilation of the vessel lumen (50 % larger than the adjacent reference vessel) at late angiography, closely related to the underlying DES or its edges [2, 10].

### OCT imaging acquisition and analysis

An M3/C7 OCT system (LightLab Imaging, USA) with an automatic pullback speed of 1.5 mm/s was used in the current study. The images included the entire length of the stent and a segment of at least 5 mm extending beyond the stent edges. The OCT images were analyzed off-line by two independent doctors blind to the research. LightLab Imaging Inc. provided the software. A single OCT cross-sectional still frame from each 1 mm segment was selected for quantitative analysis throughout the entire length of the stent. The still frames were selected based on the appearance of the stent struts and the lack of OCT motion or other image artifacts. Each stent strut in the still frame was observed to determine if the strut was a malapposition or a complete coverage. If a neointimal coverage on the strut was observed, its average thickness was measured. The presence of strut malapposition and gaps between the vessel wall and strut, as well as strut uncoverage, neointimal thickness, stent area, corresponding vessel lumen area and thrombosis were noted.

### Definition of malapposition, neointimal coverage and thrombosis

Malapposition is defined as the separation of at least one stent strut from the intimal surface of the arterial wall not overlapping a side branch, and the gap between the strut and the vessel wall should exceed 150 μm [11, 12]. Complete neointimal coverage is defined as all the stent struts were covered by the visible neointimal, strut uncoverage is confirmed if no visible neointimal coverage on the strut. Thrombosis is defined as an irregular mass with dorsal shadowing protruding from the lumen [13].

### Statistical analysis

Continuous variables are expressed as the mean ± standard deviation (SD) and are compared using an independent samples *t* test. Categorical variables are the absolute number and percentage and are compared using χ^2^ statistics or the Fisher’s exact test. A *P* value < 0.05 was considered statistically significant. Statistical evaluation was performed using a dedicated software (SPSS 11.5 for windows, SPSS, Chicago, IL, USA).

## Results

Follow-up coronary angiography was conducted on 1,160 patients who had undergone PCI, there were 56 patients occurred CAA formation at the segment of stent implantation in the period of follow-up, the ratio of CAA was 4.83 % (56/1160). Patients with CAA formation after DES implantation had a higher frequency of cardiac events, total of 40 patients occurred angina pectoris or acute myocardial infarction,the frequency of cardiac events was 71.43 % (40/56). OCT was performed in 175 patients, Table 1 shows the baseline characteristics of CAA group and non-CAA group. The demographic baseline and coronary risk factors indicates are similar between two groups except that the number of smoking patients in the CAA group is higher than that in the non-CAA group (74.19 vs. 49.92 %, *P* = 0.008). The period of follow-up was longer in the CAA group compared with the non-CAA group (24.68 ± 13.29 vs. 17.72 ± 12.71 m, *P* = 0.007). The cardiac events were frequently occurred in CAA group, there were 21 patients (10 in the non-CAA group and 11 in the CAA group) occurred angina pectoris (25.81 vs. 6.25 %, *P* = 0.001) and acute myocardial infarction (9.68 vs. 0.13 %, *P* = 0.002) during follow-up, in addition, more thrombosis were detected in CAA group (0.69 vs. 16.13 %, *P* < 0.001).

**Table 1:** Baseline characteristics

	non-CAA group	CAA group	*P* value
Patients (n)	144	31	–
Age (years)	60.44 ± 10.20	60.39 ± 12.02	0.978
Proportion of man (n, %)	115 (79.86)	23 (74.19)	0.483
Diabetes mellitus (n, %)	55 (38.19)	6 (19.35)	0.046
Hypertension (n, %)	97 (67.36)	21 (67.74)	0.967
Hypercholesterolemia (n, %)	39 (27.08)	7 (22.58)	0.605
Smoking (n, %)	69 (49.92)	23 (74.19)	0.008
Systolic blood pressure (mmHg)	129.73 ± 16.91	127.35 ± 18.33	0.486
Diastolic blood pressure (mmHg)	75.74 ± 10.62	75.29 ± 11.11	0.831
Blood glucose (mmol/L)	6.51 ± 2.19	5.44 ± 1.32	0.140
CHO (mmol/L)	3.85 ± 0.95	3.91 ± 0.49	0.867
HDL-C (mmol/L)	1.1 ± 0.26	1.1 ± 0.17	0.994
LDL-C (mmol/L)	2.19 ± 0.87	2.16 ± 0.49	0.802
TG (mmol/L)	1.83 ± 1.42	1.39 ± 0.49	0.346
Target vessel	167	33	–
RCA (n, %)	47 (28.1)	7 (21.2)	0.412
LAD (n, %)	81 (48.5)	23 (69.7)	0.026
LCX (n, %)	39 (23.4)	3 (9.1)	0.066
Stent number (n)	1.35 ± 0.56	1.52 ± 0.67	0.162
Stent diameter (mm)	3.11 ± 0.39	3.06 ± 0.31	0.509
Stent length (mm)	33.32 ± 15.54	38.81 ± 18.85	0.088
Stent types			
Cypher/Cypher select (n, %)	39 (27.08)	12 (38.71)	0.196
Endeavor (n, %)	25 (17.37)	3 (9.68)	0.29
Partner (n, %)	21 (14.58)	3 (9.68)	0.437
Firebird (n, %)	26 (18.05)	10 (32.25)	0.076
Excel (n, %)	33 (22.92)	3 (9.68)	0.098
Diagnosis in follow-up			
Angina pectoris (n, %)	9 (6.25)	8 (25.81)	0.001
Acute myocardial infarction (n, %)	1 (0.69)	3 (9.68)	0.002
Thrombosis (n, %)	1 (0.69)	5 (16.13)	<0.001
Duration between 1st and 2nd angiography (months)	17.72 ± 12.71	24.68 ± 13.29	0.007

**Fig. 1:**
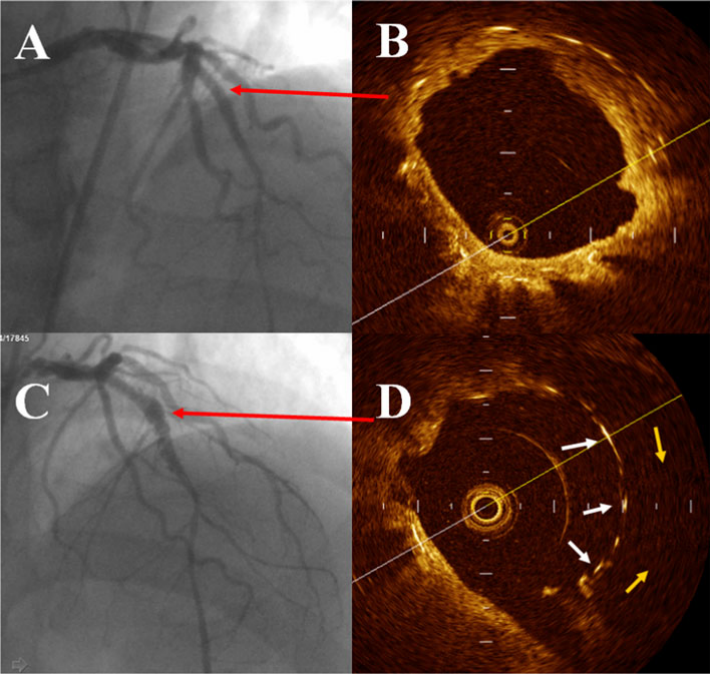
A patient with coronary aneurysm formation

OCT examination was fulfilled after coronary angiography without complication. OCT readily detected irregular vessel lumen and malapposition with a prominent cavum backside the stent struts (Fig. 1d). The optical coherence tomography results (Table 2) indicate a higher proportion of uncoverage (17.17 vs. 1.9 %, *P* < 0.001) and malapposition struts (18.2 vs. 1.38 %, *P* < 0.001) in the CAA group compared with the non-CAA group. In addition, the neointimal thickness in the CAA group is significantly thinner than non-CAA group (146.6 ± 94.8 vs. 192.5 ± 97.1 μm, *P* < 0.001), as detected by OCT. Patients with cardiac events is also analyzed as a subgroup. Table 3 shows the results of cardiac events in CAA subgroup. The longitudinal length of CAA in event group is significantly longer than in no event group (20.0 ± 9.07 vs. 12.05 ± 5.38 mm, *P* = 0.005). The percentage of stent malapposition (25.69 ± 19.01 vs. 12.77 ± 7.36 %, *P* = 0.003) and incomplete neointimal coverage (20.82 ± 17.39 vs. 12.43 ± 8.24 %, *P* = 0.027) in the group with cardiac events is significantly higher than in the no cardiac events group. There were 8 patients with cardiac events in CAA group underwent OCT examination twice in the period of follow-up. Total of 1,577 struts were detected and compared between the first and second OCT results. The proportion of incomplete neointimal coverage increased significantly as compared the second OCT results with the first examination (18.45 vs. 2.66 %, *P* < 0.001). The hyperplasia neointimal desquamated from struts and acquired struts incomplete neointimal coverage were detected, just as Fig. 1 showed.

**Table 2:** Optical coherence tomography analysis

	non-CAA group	CAA group	*P* value
Patients (n)	144	31	–
Total stent struts (n)	20969	5375	–
Uncovered stent struts (n, %)	399 (1.90)	923 (17.17)	<0.001
Stent struts malapposition (n, %)	289 (1.38)	978 (18.20)	<0.001
Neointimal thickness (μm)	192.5 ± 97.1	146.6 ± 94.8	<0.001

**Table 3:** Cardiac events in CAA subgroup

Clinical Outcome	Event	No event	*P* value
Patients (n)	11	20	–
Age (years)	62.45 ± 8.55	56.75 ± 10.18	0.126
Duration between 1st and 2nd angiography (months)	22.64 ± 13.90	25.80 ± 13.17	0.535
Aspirin treatment (n, %)	11 (100)	20 (100)	–
Aspirin and clopidogrel (n, %)	3 (27.27)	7 (35)	0.66
Stent length (mm)	45.09 ± 16.71	35.35 ± 19.47	0.173
Stent diameter (mm)	3.21 ± 0.27	2.98 ± 0.31	0.05
Longitudinal length of CAA (mm)	20.0 ± 9.07	12.05 ± 5.38	0.005
Incomplete neointimal coverage (%)	20.82 ± 17.39	12.43 ± 8.24	0.027
Stent malapposition (%)	25.69 ± 19.01	12.77 ± 7.36	0.003

A. LAD coronary angiography without aneurysm
B. OCT imaging showed stent complete neointimal coverage and well apposition
C. LAD coronary angiography with aneurysm formation
D. OCT imaging showed stent incomplete neointimal coverage(white arrow) and malapposition with cavum behind struts (yellow arrow). The bare struts had been completely coveraged by hyperplasia neointimal, as Fig. 1b showed.

## Discussion

The main findings of the current analysis are as follows: (1) CAA formation after DES implantation increases the frequency of stent malapposition and incomplete neointimal coverage,acquired incomplete neointimal coverage associated with CAA formation. (2) CAA was associated with cardiac events including angina pectoris and acute myocardial infarction.

Owing to the varying clinical presentations, CAA are usually detected at the time of repeat angiography for recurrent symptoms or the routine angiographic follow-up according to the clinical research protocols. IVUS not only readily detects the stent malapposition with a prominent distance between the struts and the vessel wall but also measures the external elastic lamina area and volume. According to the IVUS research, CAA are frequently associated with adverse clinical events as a result of DES restenosis and thrombosis [6]. However, the neointimal coverage was not assessed in IVUS clinical trials because IVUS could not clearly and accurately detect neointimal coverage. Owing to the greater spatial resolution, OCT could assess the neointimal coverage and apposition accurately after DES implantation, and stent apposition and neointimal coverage may be a useful surrogate parameter for late stent thrombosis and stent safety [14, 15]. OCT studies have reported that DES implantation for ST elevation myocardial infarction had a higher frequency of stent malapposition and incomplete neointimal coverage during follow**-**up [16, 17], but lacking of datum from CAA formation after DES implantation. In the current study, the stent malapposition and neointimal coverage in patients with CAA formation were assessed using OCT, and a higher proportion of struts incomplete neointimal coverage and malapposition was found. Struts incomplete neointimal coverage and malapposition indicates the poor vessel healing after DES implantation and as the high fisk of stent thrombosis [18]. In the research, we found stent incomplete neointimal coverage and malapposition at the segment of CAA formation, however, it was complete neointimal coverage and well apposition before CAA formation as OCT detected at first time. The procedure of aneurysms formation usually goes with a tension to stent struts and its hyperplasia intima, therefore, the hyperplasia neointimal desquamated from stent struts and the stent struts was bare again. Maybe this was a new mechanism of incomplete neointimal coverage in patients with CAA formation, but further research should be done to prove it.

Moreover, the neointimal thickness is significantly thin in patients with CAA formation. The potential mechanism is that the neointimal hyperplasia was depressed when the stent struts does not come into contact with the vessel wall. Clinical trials have indicated that stent malapposition and incomplete coverage are associated with late stent thrombosis [19, 20]. In our study, a higher number of patients with stent malapposition and incomplete neointimal coverage in the CAA group had acute myocardial infarction or angina pectoris compared with the non-CAA group. This result shows that CAA formation after DES implantation increases the cardiac events in the long-term of follow-up.

An additional phenomenon that has been observed in patients with CAA formation is the irregular vessel lumen with cavum backside the stent struts. The vessel remodeling can lead to the changement of hydromechanics and create blood whirlpool in the cavum at the segment of CAA. This is also a risk factor of stent thrombosis. We also find that cardiac events readily occurred in the patients with the longer longitude of CAA. It indicates the more diffused CAA formation the more risk of cardiac events. The average time between the first and second coronary angiography in CAA group is (24.68 ± 13.29) months, most patients undertook single Asprin to antiplatelet therapy before CAA was diagnosed because dual antiplatelet therapy was modified after 1 year duration. Notwithstanding some patients with CAA formation without clinical symptoms, they still belong to the high-risk group of cardiac events. Therefore, lifelong dual antiplatelet therapy such as aspirin and clopidogrel, is necessary for those patients in order to reduce the risk of stent thrombosis. However, there is no consensus regarding the CAA treatment, further study is needed to assess the strategy.

The mechanism of CAA is still unclear even though previous reports described local hypersensitivity and chronic inflammation [3, 4]. The polymer carrier of DES also induced the inflammatory reaction of the arterial wall [21]. CAA formation is a slowly Pathophysiologic procedure, it is important to detect CAA at early stage. Late positive remodeling after stent implantation had been assessed by IVUS and late stent malapposition was also observed frequently, although the relationship between CAA and late stent malapposition remains to be determined. Hong et al. [22] reported that the predictors of late stent malapposition include the total stent length, primary stenting in acute myocardial infarction, and chronic total occlusion lesions; however, all factors are correlated with the characteristics of lesion and PCI. Smoking has been proven a risk factor for cardiovascular disease and is associated with restenosis and late thrombosis [23, 24]. Smoking can induce an endothelium function disorder as well as chronic inflammation around the stent, and the vessel wall responded to the pathological procedure. The result also indicates the importance of smoking cessation in patients who underwent DES implantation.

## Limitations

The current study has several limitations. First, it is a single-center study that involved a small sample. A study involving larger patient populations from various centers is warranted to confirm the results. Second, the limited penetration of the current OCT cannot accurately detect the components of deep structures, especially the external elastic membrane of the vessel, hence the whole profile of the cavum backside stent is not clear. In addition, OCT cannot detect a very thin intimal coverage (<10 μm) although it was the highest resolution technique at the present time. This maybe increase the frequency of uncovered stent struts during OCT imaging analysis.

## Conclusions

CAA formation after DES implantation frequently associated with cardiac events as a result of stent malapposition and incomplete neointimal coverage. Acquired incomplete neointimal coverage associated with CAA formation.
